# Bruceine A attenuates renal tubulointerstitial fibrosis by inhibiting the Wnt7a/PI3K/AKT pathway

**DOI:** 10.3389/fphar.2026.1865261

**Published:** 2026-09-07

**Authors:** Shuangyu Li, Renqing Wang, Tong Wang, Wenyan Zhang, Shengchun Xu, Zhenhua Ren, Renfei Luo

**Affiliations:** 1 Department of Anatomy, School of Basic Medicine, Anhui Medical University, Hefei, Anhui, China; 2 Department of Pathology, The First Affiliated Hospital of Anhui Medical University, Hefei, Anhui, China

**Keywords:** AKT, Bruceine A, PI3K, tubulointerstitial fibrosis, Wnt7a

## Abstract

**Introduction:**

Tubulointerstitial fibrosis (TIF) is a major pathological feature of progressive chronic kidney disease (CKD), yet effective pharmacological therapies remain limited. Bruceine A (BA), a quassinoid isolated from *Brucea javanica*, has been reported to exert renoprotective effects in glomerular diseases. However, its pharmacological role in renal TIF and the underlying mechanisms remain poorly defined.

**Methods:**

In this study, we investigated the effects of BA in two classic animal models of TIF, unilateral ureteral obstruction (UUO) and unilateral ischemia-reperfusion injury (UIRI), as well as in TGF-β1-stimulated HK-2 cells.

**Results:**

BA significantly attenuated renal histopathological injury, inflammatory responses, and fibrotic changes in both in vivo models, and improved renal function in UIRI mice. BA dose-dependently suppressed TGF-β1-induced epithelial-mesenchymal transition (EMT) and fibrotic responses in HK-2 cells. Mechanistically, RNA sequencing identified Wnt7a as a BA-responsive gene in fibrotic kidneys. BA consistently reduced Wnt7a expression in vivo and in vitro, while integrated RNA-seq and network pharmacology analyses implicated PI3K/AKT signaling as a key downstream pathway, which was further supported by reduced PI3K and AKT phosphorylation after BA treatment. Moreover, recombinant Wnt7a protein and the AKT agonist SC79 partially reversed BA-mediated suppression of fibrotic marker expression in HK-2 cells. Recombinant Wnt7a also partially restored PI3K/AKT activation.

**Discussion:**

Collectively, these findings suggest that BA attenuates renal tubulointerstitial injury and fibrosis, potentially through modulation of the Wnt7a/PI3K/AKT signaling pathway, supporting its potential as a therapeutic candidate for TIF.

## Introduction

Chronic kidney disease (CKD) is increasingly recognized as a global public health problem, contributing to significant morbidity and mortality, and financial strain on healthcare systems (Cockwell and Fisher, 2020; Herrington et al., 2026). Renal tubulointerstitial fibrosis (TIF), the final common outcome of almost all progressive CKDs regardless of the original etiologies, is characterized by epithelial-to-mesenchymal transition (EMT) of renal tubular epithelial cells (TECs) and excessive deposition of extracellular matrix (ECM) (Zhou and Liu, 2016). TIF involves complex interactions between TECs, myofibroblasts, endothelial cells, and inflammatory cells (Zhou et al., 2019; Chen et al., 2024; Xiao et al., 2025). Whereas mechanisms underlying TIF have grown in scope and understanding in recent decades, effective treatment to directly halt or even reverse fibrosis remains limited. Therefore, better understanding of these mechanisms and development of potential drugs for TIF are urgently needed.

Bruceine A (BA) is a quassinoid compound derived from the fruit of *Brucea javanica*, known for extensive and distinctive pharmacological activities including anti-parasitic, antitumor, and anti-fibrotic effects (Wang et al., 2011; Zhang et al., 2021; Sun et al., 2025). In kidney disease research, recent studies have successively reported that BA protected common types of glomerular diseases, including diabetic nephropathy (DN) and mesangial proliferative glomerulonephritis (MsPGN) (Li et al., 2022; He et al., 2025; Hu et al., 2025). Mechanistically, BA exerts its protective effects by suppressing inflammatory and proliferative responses of glomerular mesangial cells. However, whether BA exerts a direct protective effect against renal tubulointerstitial injury and fibrosis in CKD remains largely unknown.

Wnt7a, a member of the Wnt ligand family, has emerged as an important pro-fibrotic mediator in idiopathic pulmonary fibrosis and cardiac fibrosis (Konigshoff and Eickelberg, 2023; Yuan et al., 2024). In the kidney, Wnt7a is upregulated in experimental models such as unilateral ureteral obstruction (UUO), and aberrant Wnt7a signaling has also been reported in polycystic kidney disease (PKD), supporting a potential role in chronic kidney injury and remodeling (Qin et al., 2012; Ming et al., 2023). Numerous studies have also shown that inhibition of Wnt secretion or blockade of Wnt/β-catenin signaling can alleviate renal injury and fibrosis (Zhou et al., 2017; Luo et al., 2018). Therefore, targeting Wnt7a may represent a promising therapeutic strategy for tubulointerstitial injury and fibrosis.

PI3K/AKT-mediated fibrogenesis has emerged as a key driver of CKD progression and an important therapeutic target. Increasing evidence indicates that natural compounds derived from medicinal plants and dietary sources, such as formononetin and luteolin, exert renoprotective effects by modulating the PI3K/AKT signaling axis, together with related inflammatory and oxidative stress pathways, thereby attenuating renal injury and fibrotic remodeling (Singh A. et al., 2026; Singh L. et al., 2026). Although crosstalk between Wnt7a and PI3K/AKT pathway has been reported in skeletal muscle hypertrophy, wound healing, and tumorigenesis, the regulatory role of Wnt7a in the PI3K/AKT pathway during renal TIF remains unclear (von Maltzahn et al., 2011). Therefore, Wnt7a may be more appropriately regarded as a candidate upstream profibrotic ligand whose interaction with PI3K/AKT signaling in renal fibrosis warrants further investigation.

In the present study, we aimed to evaluate the effect of BA on renal tubulointerstitial fibrosis using two established mouse models, UUO and UIRI, and to investigate the involvement of the Wnt7a/PI3K/AKT pathway in its anti-fibrotic effect.

## Materials and methods

### Drug information and preparation

Bruceine A (BA; purity 96%, 5 mg per vial; product No. B860733) was purchased from Shanghai Macklin Biochemical Technology Co., Ltd. For *in vivo* studies, BA was dissolved in anhydrous ethanol to prepare a 10 mg/mL stock solution, aliquoted, and stored at −20 °C in the dark for up to 1 week. On each dosing day, the stock was diluted with ethanol and saline to the required concentration according to body weight and target dose. The vehicle consisted of 5% ethanol in normal saline, and BA was administered via intraperitoneal injection at 0.25 mL per 25 g body weight. For *in vitro* study, BA (5 mg) was dissolved in DMSO (1 mL) to obtain a 10 mM stock solution and stored at −20 °C in the dark. A 5 μM working solution was prepared by dilution in complete medium (final DMSO 0.05%). HK-2 cells were treated with BA by adding appropriate volumes of working solution to 1 mL medium per well (final DMSO 0.002%). Vehicle controls received equivalent DMSO.

### Animals and treatments

Male C57BL/6J mice aged 8 weeks and weighing 22–24 g were purchased from Jiangsu GemPharmatech Co., Ltd. Mice were maintained in a temperature-controlled barrier facility under a 12:12-h light/dark cycle and were provided free access to standard laboratory chow and tap water. All animal experiments were approved by the Animal Ethics Committee of Anhui Medical University (Approval No. LLSC20231900; approval date: May 20, 2023) and were conducted in accordance with institutional guidelines for the care and use of laboratory animals. Protocol I: Mice were randomly divided into four groups: (1) Sham operation + vehicle; (2) UUO + vehicle; (3) UUO + BA low dose, 0.5 mg/kg body weight/day; and (4) UUO + BA high dose, 1 mg/kg body weight/day. UUO surgery was performed as previously described (Luo et al., 2017). Briefly, the left ureter was ligated with 3–0 surgical suture. Vehicle or BA was administered immediately after Sham operation or UUO surgery on day 0 and continued once daily for 7 consecutive days. Mice were euthanized on day 7 for serum and tissue collection. Protocol II: Mice were randomly divided into three groups: (1) Sham operation + vehicle; (2) UIRI + vehicle; and (3) UIRI + BA, 1 mg/kg body weight/day. The renal UIRI model was established as previously described (Li et al., 2017). Briefly, under general anesthesia, a midline abdominal incision was made, and the left renal pedicle was clamped for 35 min using a vascular clamp. Mice were maintained on a heating pad at 37 °C during ischemia. Vehicle or BA was administered immediately after Sham operation or UIRI surgery on day 0 and continued once daily for 14 consecutive days. To minimize the compensatory influence of the contralateral kidney on renal function assessment, the right kidney was surgically removed on day 13 after ischemia–reperfusion. Mice were euthanized on day 14 for serum and tissue collection.

### Kidney weight assay

After spontaneous urination, body weight was measured using a precision electronic balance. Fresh kidneys were collected and rinsed three times with pre-chilled saline. Surface moisture was removed with filter paper, and the renal capsules were carefully stripped on ice. The kidneys were then weighed immediately. Corrected kidney weight was calculated as the ratio of kidney weight to body weight and expressed as mg/g.

### Assessment of renal function

Blood samples were collected from anesthetized mice by puncturing the inferior vena cava using a 1-mL insulin syringe containing 50 μL of 1 mM EDTA. Serum was obtained by centrifugation at 1000 rpm for 10 min. Serum creatinine (SCr) levels were measured using a commercial creatinine assay kit (Nanjing Jiancheng Bioengineering Institute, C011-2-1) according to the manufacturer’s instructions, and absorbance was determined using a microplate reader. Serum blood urea nitrogen (BUN) levels were measured using a BUN assay kit (Nanjing Jiancheng Bioengineering Institute, C013-3-1) following the manufacturer’s protocol.

### Histology and immunohistochemistry

Paraffin-embedded kidney sections (4 μm thickness) were prepared using standard procedures. Sections were stained with hematoxylin and eosin (H&E) for general histopathological evaluation and with Masson’s trichrome staining (MTS) to assess collagen deposition and fibrotic lesions. Tubular injury was scored based on the percentage of injured tubules as follows: 0, no damage; 1, < 25%; 2, 25%–50%; 3, 50%–75%; 4, > 75%. Fibrotic areas were quantified using ImageJ software and expressed as the percentage of Masson-positive area relative to the total tissue area. At least three randomly selected fields were analyzed per mouse.

### Immunofluorescence staining

The tissues were fixed in 10% neutral buffered formalin for 24 h and then, embedded in paraffin. After deparaffinization, thin sections (4 μm) were processed for double labeling with immunofluorescence. The slides were blocked in 1% BSA for 1 h. The primary antibodies used were as follows: anti-FN1, 1:400 dilution (BOSTER, BA1772); anti-α-SMA, 1:400 dilution (BOSTER, BM0002); anti-COL1A1, 1:200 dilution (BOSTER, BA0325). After washing off the primary antibody, sections were incubated for 1 h at room temperature goat anti rabbit IgG-AbFluor-488 (Immunoway, RS3211). Nuclei were stained with DAPI (Immunoway, YS0014) according to the manufacturer’s instructions. Images were captured using Zeiss LSM 980 confocal microscope (Carl Zeiss Microscopy GmbH, Jena, Germany).

### Cell culture and treatment

Human proximal tubular epithelial cells (HK-2 cells) were obtained from Shanghai Fuheng Biotechnology Co., Ltd. and cultured in DMEM/F12 (Thermo Fisher Scientific) supplemented with 10% fetal bovine serum (FBS; Thermo Fisher Scientific) and 1% penicillin/streptomycin (Thermo Fisher Scientific) at 37 °C in a humidified atmosphere containing 5% CO_2_. Cells were seeded in 6-well plates (Thermo Fisher Scientific) at a density of 2 × 10^5^ cells/well and cultured for 24 h, followed by serum starvation for an additional 24 h. Protocol I: Effects of BA on TGF-β1-induced fibrotic response. Following serum starvation, HK-2 cells were randomly divided into the following groups: (1) control group (CTL); (2) TGF-β1 group, treated with recombinant human TGF-β1 (10 ng/mL; MedChemExpress) for 48 h; and (3) TGF-β1 + BA groups, co-treated with TGF-β1 (10 ng/mL) and BA at 50, 100, or 200 nM (Shanghai Macklin Biochemical Co., Ltd.) for 48 h. Protocol II: Rescue experiments with WNT7A or SC79. To further investigate the involvement of the PI3K/AKT signaling pathway in the effects of BA, rescue experiments were performed. HK-2 cells were co-treated with TGF-β1 (10 ng/mL) and BA (100 nM) as described above, followed by supplementation with either recombinant WNT7A (5 ng/mL; MedChemExpress) or SC79 (10 μM; an AKT agonist; MedChemExpress) for 48 h. After treatment, cells were harvested for subsequent protein and RNA analyses.

### Western blot

Western blot analysis was carried out as previously described. Briefly, 30 μg of protein for each sample was denatured in a boiling water bath for 10 min, separated by SDS-PAGE, and transferred to nitrocellulose membrane (Immobilion-P; Millipore, Bedford, MA). The membranes were blocked with 5% nonfat dry milk in Tris-buffered saline with Tween 20 (TBST) for 1 h at room temperature followed by incubation with primary antibodies [ FN1, 1:3000 dilution (BOSTER, BA1772); α-SMA, 1:3000 dilution (BOSTER, BM0002); COL1A1, 1:1000 dilution (BOSTER, BA0325); α-Tubulin, 1:5000 dilution (Proteintech, 11224-1-AP); Wnt7a, 1:1000 dilution (Proteintech, 10605-1-AP); AKT, 1:2000 dilution (Abways, CY5561); p-AKT, 1:1000 dilution (Abways, CY6569); PI3K, 1:2000 dilution (Abways, CY5355); p-PI3K, 1:1000 dilution (Abways, CY6427); GAPDH, 1:5000 dilution (BOSTER, A00227-1) ] at 4 °C for overnight. After being washed with TBST, membranes were incubated with secondary antibodies (goat anti-rabbit horseradish peroxidase-conjugated secondary antibody; BOSTER) for 1 h at room temperature and visualized with enhanced chemiluminescence (Thermo Scientific). The blots were visualized in an ECL detection system (Thermo Scientific) and densitometric analysis was performed using ImageJ software.

### Real-time PCR

Snap-frozen renal samples were homogenized in TRIzol reagent (Beyotime, R0016). Total RNA isolation and reverse transcription were performed as previously described. Total RNA concentrations were determined using a NANODROP 2000 spectrophotometer (Thermo Scientific) according to the manufacturer’s instructions. We used 1 μg of total RNA as a template for RT by using SPARK script II All-in-one RT SuperMix for qPCR (SparkJade, AG0305-B) according to the manufacturer’s instructions. Real-time quantitative PCR was performed using 2 × SYBR Green qPCR Mix (SparkJade, AH0104-B) according to the manufacturer’s instructions. Relative mRNA expression levels were calculated from threshold cycle numbers (CT), i.e., 2^−ΔΔCT^, according to the manufacturer’s suggestion. The data were shown as a relative value normalized by GAPDH. The primers were synthesized by Tsingke Biotech, and the sequences were listed in Supplementary Table S1.

### Network pharmacological analysis

Acquisition of BA-related targets: The SMILES format of BA was retrieved from the PubChem database and imported into the SwissTargetPrediction platform (http://swisstargetprediction.ch/, accessed on 21 July 2025) for potential target prediction. Acquisition of renal tubulointerstitial fibrosis-associated genes: The GeneCards database (version 2024, https://www.genecards.org/) was searched using the keyword “renal tubulointerstitial fibrosis”, and genes with a relevance score ≥ 10 were selected. The GeneCards database (version 2024, https://www.genecards.org/, accessed on 21 July 2025) was searched using the keyword “renal tubulointerstitial fibrosis”, and genes with a relevance score ≥ 10 were selected. Intersection target screening: The BA targets and disease targets were uploaded to the Venn diagram online tool (Venny 2.1, https://bioinfogp.cnb.csic.es/tools/venny/) to obtain the overlapping targets. Protein–protein interaction (PPI) network analysis: The STRING database (version 11.5, https://string-db.org/) was used with the species set to *Homo sapiens* and a confidence score ≥ 0.4 as the threshold to construct the PPI network. Cytoscape (version 3.7.2) with the cytoHubba plugin was employed to rank hub genes using the Maximal Clique Centrality (MCC) algorithm; the top 10 hub genes were selected. Enrichment analysis: The intersecting targets were subjected to KEGG pathway enrichment analysis using the Metascape platform (https://metascape.org/). The significance threshold was set at P < 0.01, with a minimum count of 3 and an enrichment factor > 1.5.

### RNA sequencing and data analysis

Total RNA was extracted from mouse kidney tissues using TRIzol reagent (Invitrogen). RNA quality and integrity were assessed using an Agilent 2100 Bioanalyzer. Sequencing libraries were generated following mRNA enrichment with poly-T oligo–attached magnetic beads and subsequent cDNA synthesis. Library quality was evaluated using the Agilent Bioanalyzer 2100 system. Libraries were sequenced on an Illumina NovaSeq platform (Chengqi Biotechnology, Guangzhou, China) to generate 150-bp paired-end reads. Raw reads were filtered to remove adaptor sequences and low-quality reads using in-house Perl scripts. Clean reads were aligned to the reference genome using HISAT2 (v2.0.5). Gene expression levels were quantified using FeatureCounts, and normalized as FPKM values. Differential expression analysis was performed using DESeq2 (v1.20.0). Genes with adjusted P < 0.05 were considered differentially expressed. Functional enrichment analyses, including GO, KEGG, and Reactome, were conducted using the clusterProfiler R package. Gene set enrichment analysis (GSEA) was performed to identify enriched pathways based on GO, KEGG, Reactome, Disease Ontology (DO), and DisGeNET gene sets.

### Statistical analysis

Data are summarized as means ± SD. Normality was assessed using the Shapiro–Wilk test, and homogeneity of variance was evaluated using Levene’s test. For normally distributed data with homogeneous variance, comparisons between two groups were performed using two-tailed Student’s *t-test*, while comparisons among multiple groups were performed using one-way ANOVA followed by Tukey’s *post hoc* test. A two-sided *P < 0.05* was considered statistically significant.

## Results

### BA attenuated renal histopathological injury and TIF in UUO mice

**FIGURE 1:**
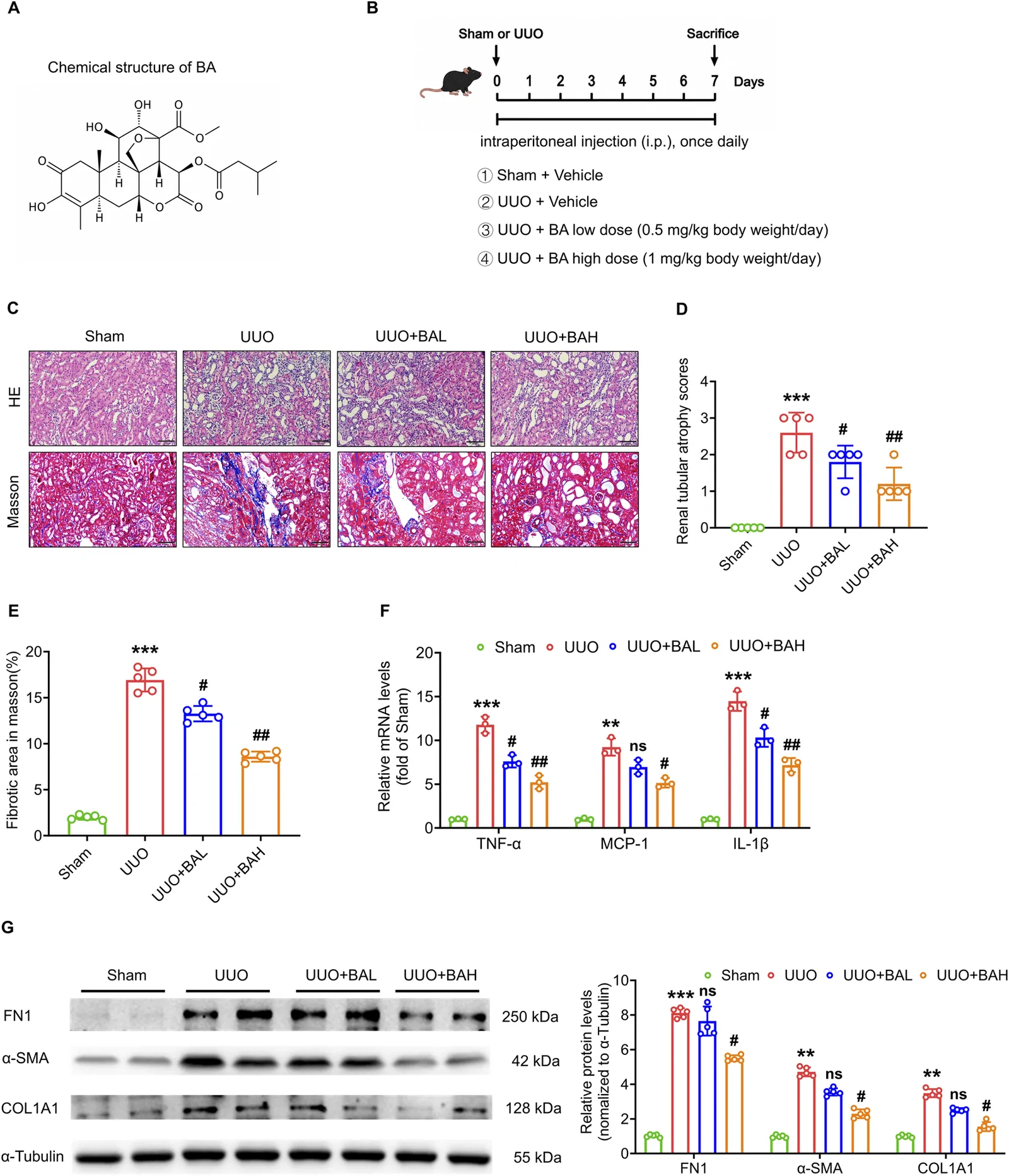
BA ameliorated unilateral ureteral obstruction (UUO)-induced renal fibrosis and inflammation in mice. **(A)** Chemical structure of BA. **(B)** Schematic diagram of the experimental design. Detailed experimental procedures are described in the Methods section. **(C)** Representative hematoxylin and eosin (H&E) staining and Masson’s trichrome staining of kidney sections from each experimental group. Scale bar, 100 μm. **(D)** Quantification of renal tubular injury scores based on H&E staining. **(E)** Quantification of renal fibrosis area based on Masson’s trichrome staining. **(F)** mRNA expression levels of tumor necrosis factor-α (TNF-α), monocyte chemoattractant protein-1 (MCP-1), and interleukin-1β (IL-1β) in kidney tissues were measured by RT–qPCR. **(G)** Representative western blot images and quantitative analysis of fibronectin (FN), α-smooth muscle actin (α-SMA), and collagen I (COL I) protein expression. Data shown are individual values with means ± SD; n = 3–5. *P < 0.05, significantly different from Sham group; #P < 0.05, significantly different from UUO group. ns: not significant. Error bars and exact P values are reported in Supplementary File 1.

The chemical structure of BA is shown in Figure 1A. UUO-induced mouse model of TIF was established, and the experimental protocol is illustrated in Figure 1B. H&E and Masson’s trichrome staining showed that mice in the UUO group developed marked renal tubular injury, characterized by cortical tubular dilatation and atrophy, increased interstitial inflammatory cell infiltration, and prominent collagen deposition. BA treatment partially alleviated these histopathological alterations (Figures 1C–E). Given the critical role of tubular inflammation in the progression of TIF, we next assessed the altered expression of inflammatory cytokines in obstructed kidneys. BA treatment significantly reduced the mRNA levels of TNF-α, MCP-1, and IL-1β compared with those in the UUO group (Figure 1F). In addition, the UUO-induced upregulation of fibrosis-related proteins, including FN1, Col 1A1, and α-SMA, was markedly suppressed by BA treatment (Figure 1G). Taken together, these findings indicate that BA ameliorates UUO-induced renal histopathological injury, inflammation, and TIF in mice, with more pronounced protective effects observed at 1 mg/kg body weight/day than at 0.5 mg/kg body weight/day.

### BA improved renal function and attenuated histopathological injury and TIF in UIRI mice

**FIGURE 2:**
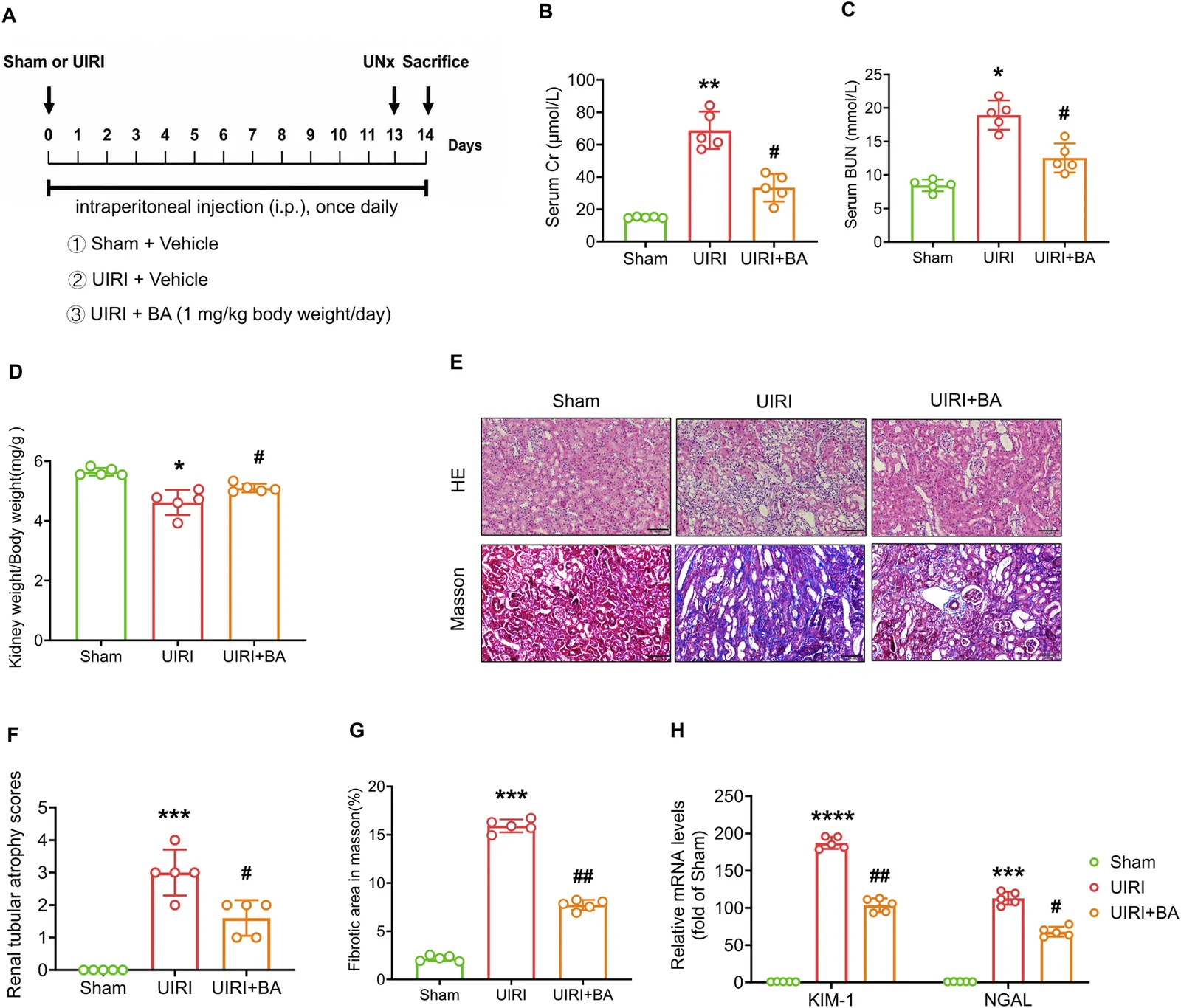
BA improved renal function and alleviated kidney injury in mice subjected to unilateral ischemia-reperfusion injury (UIRI). **(A)** Schematic diagram of the experimental design. Detailed experimental procedures are described in the Methods section. **(B)** Serum creatinine (Scr) levels. **(C)** Blood urea nitrogen (BUN) levels. **(D)** Kidney-to-body weight ratio in each group. **(E)** Representative images of hematoxylin and eosin (H&E) staining and Masson’s trichrome staining of kidney sections from each experimental group. Scale bar, 100 μm. **(F)** Quantitative analysis of renal tubular injury scores based on H&E staining. **(G)** Quantification of renal fibrosis area based on Masson’s trichrome staining. **(H)** mRNA expression levels of kidney injury molecule-1 (KIM-1) and neutrophil gelatinase-associated lipocalin (NGAL) in kidney tissues were measured by RT–qPCR. Data shown are individual values with means ± SD; n = 5. *P < 0.05, significantly different from Sham group; #P < 0.05, significantly different from UIRI group. Error bars and exact P values are reported in Supplementary File 1.

**FIGURE 3:**
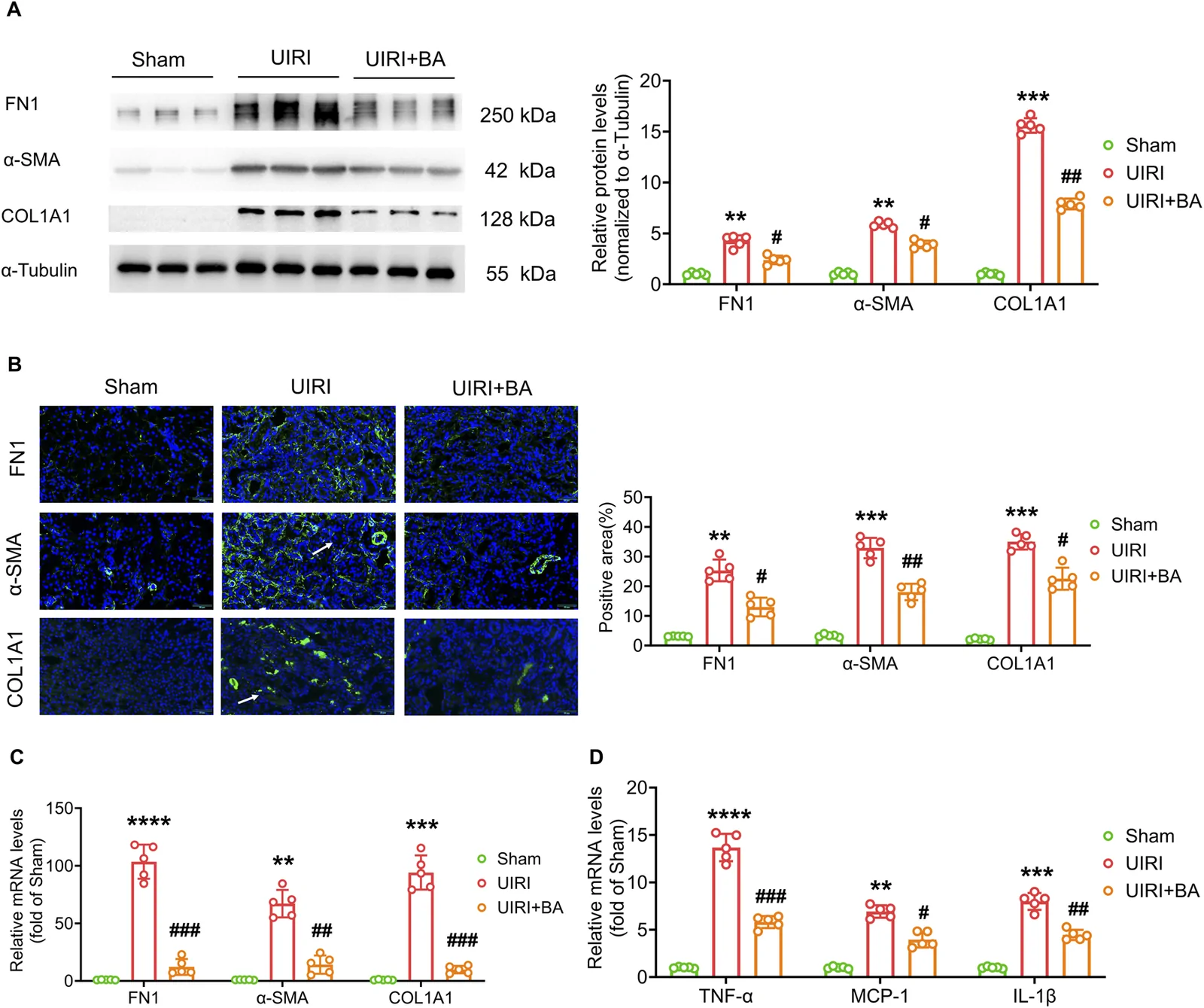
BA attenuate UIRI-induced renal fibrosis and inflammation in mice. **(A)** Representative western blot images and quantitative analysis of fibronectin (FN), α-smooth muscle actin (α-SMA), and collagen I (COL I) protein expression. **(B)** Representative immunofluorescence micrographs showing renal FN, α-SMA, and COL I expression in different groups. White arrows indicate positive staining. Scale bar, 50 μm. **(C)** mRNA expression levels of FN, α-SMA, and COL I in kidney tissues were measured by RT–qPCR. **(D)** mRNA expression levels of tumor necrosis factor-α (TNF-α), monocyte chemoattractant protein-1 (MCP-1), and interleukin-1β (IL-1β) in kidney tissues were measured by RT–qPCR. Data shown are individual values with means ± SD; n = 5. *P < 0.05, significantly different from Sham group; #P < 0.05, significantly different from UIRI group. Error bars and exact P values are reported in Supplementary File 1.

To further validate the protective effect of BA on TIF, we employed another well-established TIF model induced by UIRI. The animal experimental design is shown in Figure 2A. UIRI markedly increased serum creatinine (Scr) and blood urea nitrogen (BUN) levels compared with the sham group, whereas BA treatment significantly attenuated these changes (Figures 2B,C). A reduction in kidney weight is considered a macroscopic indicator of progressive renal fibrosis during the transition from acute kidney injury (AKI) to CKD. Kidney weight normalized to body weight was significantly decreased in the UIRI group and was partially restored by BA treatment (Figure 2D). Histologically, H&E and Masson’s trichrome staining demonstrated marked tubular injury and collagen deposition in UIRI kidneys, both of which were alleviated by BA treatment (Figures 2E–G). KIM-1 and NGAL, two established markers of tubular injury, were markedly upregulated at the mRNA level in the UIRI group compared with the sham group, whereas BA treatment reduced their expression (Figure 2H). In addition, western blot and densitometric analyses showed that BA significantly suppressed the UIRI-induced upregulation of fibrosis-related proteins, including FN1, Col 1A1, and α-SMA (Figure 3A), which was further supported by immunofluorescence staining (Figure 3B). Consistently, the mRNA expression of these fibrotic markers was also significantly decreased by BA treatment (Figure 3C). Furthermore, BA reduced the UIRI-induced increase in renal TNF-α, MCP-1, and IL-1β mRNA levels (Figure 3D). Together, these data demonstrate that BA exerts a protective effect against UIRI-induced renal injury, inflammation, and TIF.

### BA inhibited TGF-β1–induced epithelial-mesenchymal transition (EMT) and fibrotic responses in HK-2 cells

**FIGURE 4:**
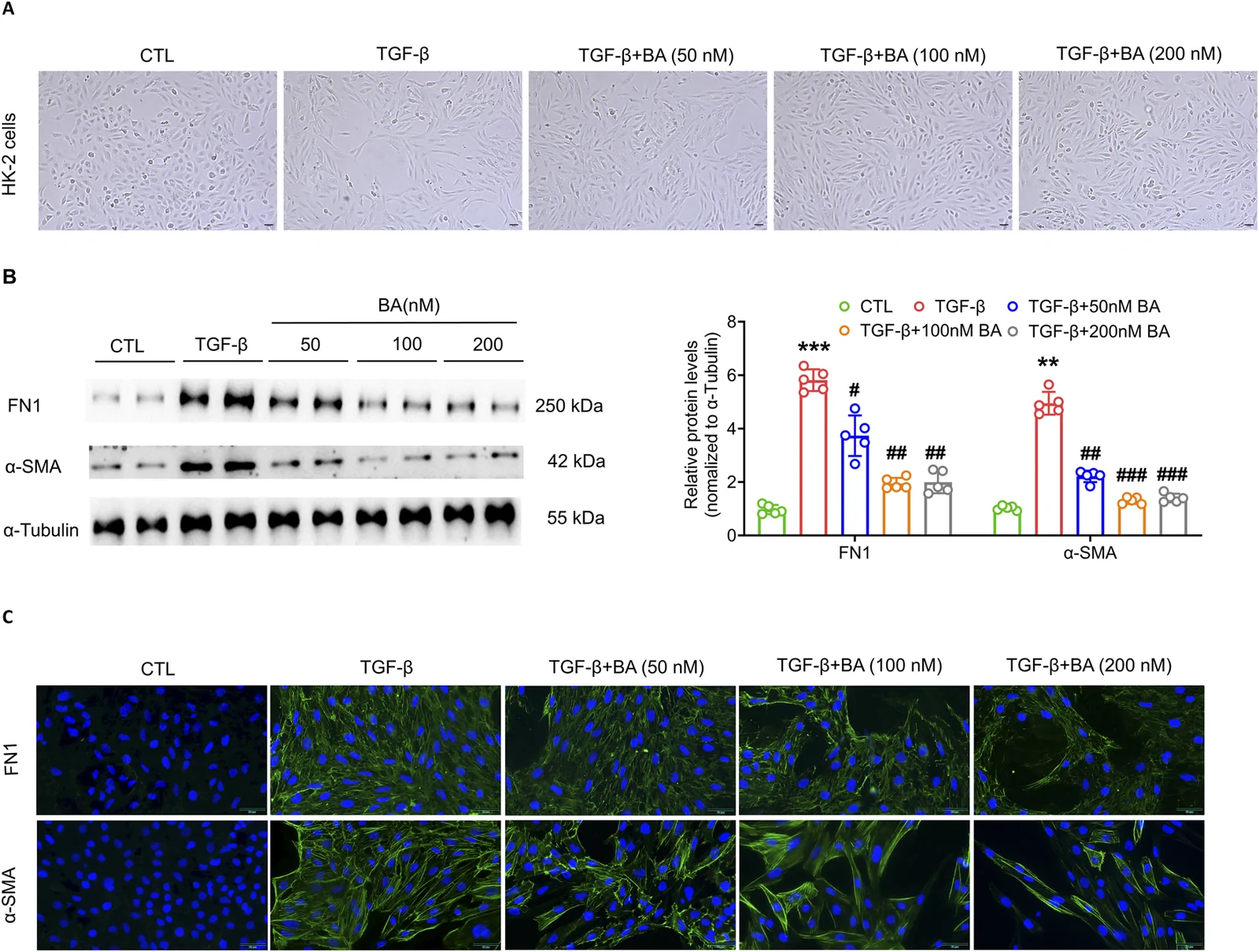
BA inhibited TGF-β1–induced epithelial-mesenchymal transition (EMT) in HK-2 cells. **(A)** Representative phase-contrast images of HK-2 cells treated with recombinant human TGF-β1 (10 ng/mL) for 48 h, with or without BA (50, 100, or 200 nM). Scale bar, 200 μm. **(B)** Representative western blot images and quantitative analysis of fibronectin (FN) and α-smooth muscle actin (α-SMA) protein expression. **(C)** Representative immunofluorescence micrographs showing FN and α-SMA expression in different groups. Scale bar, 50 μm. Data shown are individual values with means ± SD; n = 5. *P < 0.05, significantly different from CTL group; #P < 0.05, significantly different from TGF-β group. Error bars and exact P values are reported in Supplementary File 1.

To determine whether BA attenuates TGF-β1-induced EMT and fibrotic responses in HK-2 cells, we first examined cell morphology. As shown in Figure 4A, TGF-β1 stimulation induced typical EMT-like changes, characterized by loss of epithelial cobblestone morphology and acquisition of an elongated, spindle-shaped phenotype. BA treatment dose-dependently attenuated these morphological alterations and partially restored epithelial features. We next assessed the expression of fibrosis-associated proteins. Western blot analysis showed that TGF-β1 markedly increased the expression of FN1 and α-SMA, whereas BA treatment dose-dependently reduced their expression. Densitometric analysis further supported these findings (Figure 4B). Consistently, immunofluorescence staining demonstrated increased FN1 and α-SMA expression following TGF-β1 stimulation, accompanied by pronounced cytoskeletal reorganization, all of which were markedly attenuated by BA treatment (Figure 4C). Collectively, these results indicate that BA suppresses TGF-β1-induced EMT and fibrotic responses in HK-2 cells.

### BA suppressed Wnt7a expression in fibrotic kidneys from UUO and UIRI mice, as well as in TGF-β1-treated HK-2 cells

**FIGURE 5:**
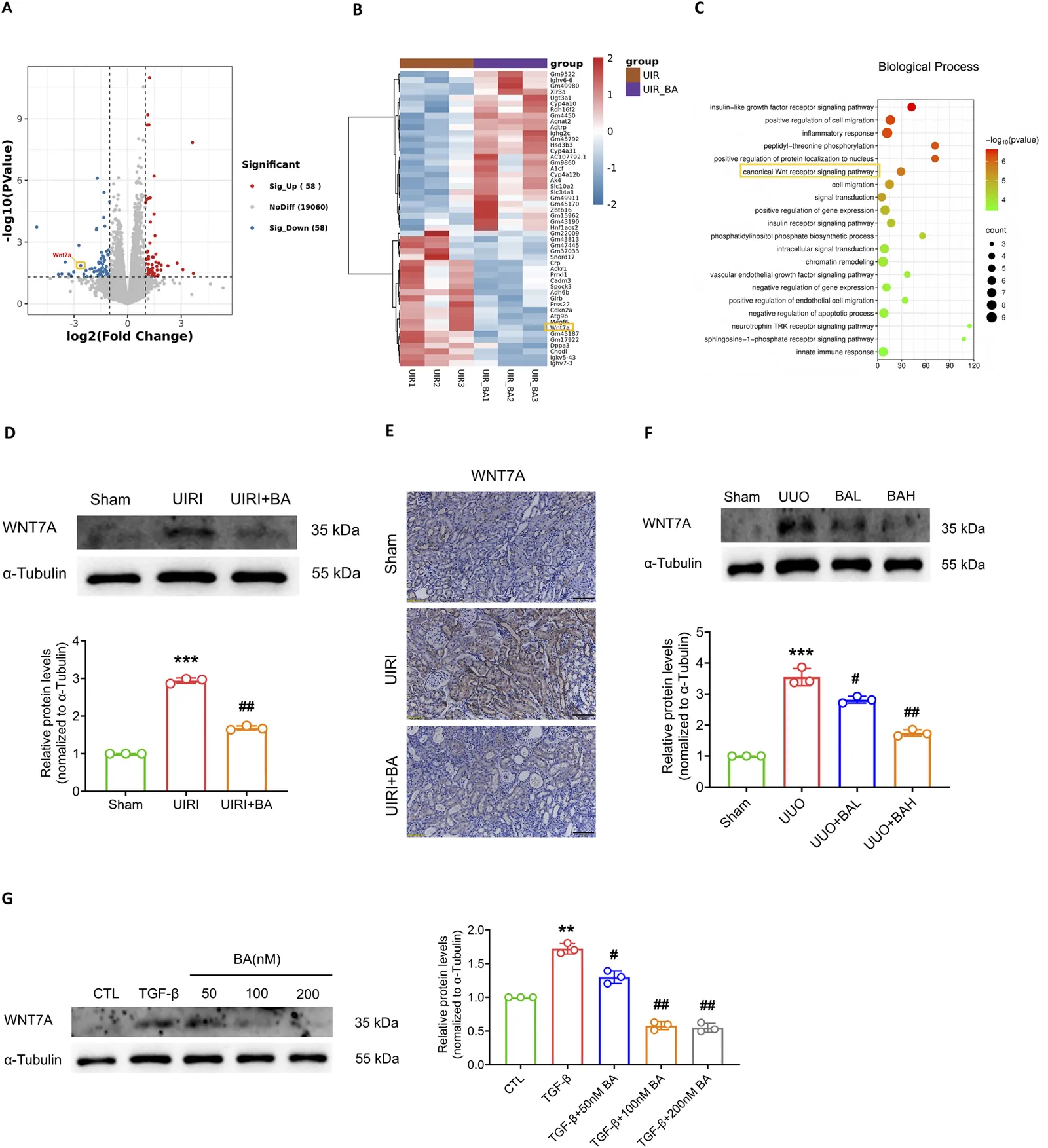
BA reversed the upregulation of Wnt7a expression in fibrotic kidneys of UIRI and UUO mice, as well as in TGF-β1-treated HK-2 cells. **(A)** Three independent biological replicates were included in the UIRI and UIRI + BA groups for RNA-seq analysis. Volcano plot showing differentially expressed genes between the UIRI and UIRI + BA groups. **(B)** Hierarchical clustering analysis of differentially expressed genes between the UIRI and UIRI + BA groups. **(C)** Gene Ontology (GO) biological process enrichment analysis of differentially expressed genes. Bubble size indicates gene count, bubble color represents −log10 (P value), and the x-axis represents gene ratio. **(D)** Representative western blot images and quantitative analysis of Wnt7a protein expression in kidneys from Sham, UIRI, and UIRI + BA groups. Data shown are individual values with means ± SD; n = 3. *P < 0.05, significantly different from Sham group; #P < 0.05, significantly different from UIRI group. **(E)** Representative immunohistochemical staining images showing Wnt7a expression in renal sections from Sham, UIRI, and UIRI + BA groups. Scale bar, 100 μm. **(F)** Representative western blot images and quantitative analysis of Wnt7a protein expression in kidneys from Sham, UUO, UUO + BA low-dose (BAL), and UUO + BA high-dose (BAH) groups. Data shown are individual values with means ± SD; n = 3. *P < 0.05, significantly different from Sham group; #P < 0.05, significantly different from UUO group. **(G)** Representative western blot images and quantitative analysis of Wnt7a protein expression in HK-2 cells treated with TGF-β1, with or without BA (50, 100, or 200 nM). Data shown are individual values with means ± SD; n = 3. *P < 0.05, significantly different from CTL group; #P < 0.05, significantly different from TGF-β group. Error bars and exact P values are reported in Supplementary File 1.

RNA sequencing analysis identified 58 upregulated and 58 downregulated genes between the UIR and UIR + BA groups. Among these, Wnt7a was significantly upregulated in the UIR group and markedly suppressed by BA treatment (Figure 5A). Heatmap analysis further demonstrated that BA reversed the overall transcriptional alterations induced by UIR (Figure 5B). Gene Ontology enrichment analysis revealed that the differentially expressed genes were primarily associated with canonical Wnt receptor signaling pathway (Figure 5C). Consistently, western blot analysis showed that Wnt7a protein expression was significantly increased in UIR kidneys compared with the sham group, whereas BA treatment markedly reduced its expression (Figure 5D). Immunohistochemical staining further confirmed these findings at the tissue level (Figure 5E). Importantly, similar results were observed in the UUO model and HK-2 cells, where BA treatment dose-dependently suppressed Wnt7a expression, supporting the reproducibility of these findings (Figures 5F,G).

### BA inhibited the activation of PI3K-AKT pathway in fibrotic kidneys from UUO and UIRI mice, as well as in TGF-β1-treated HK-2 cells

**FIGURE 6:**
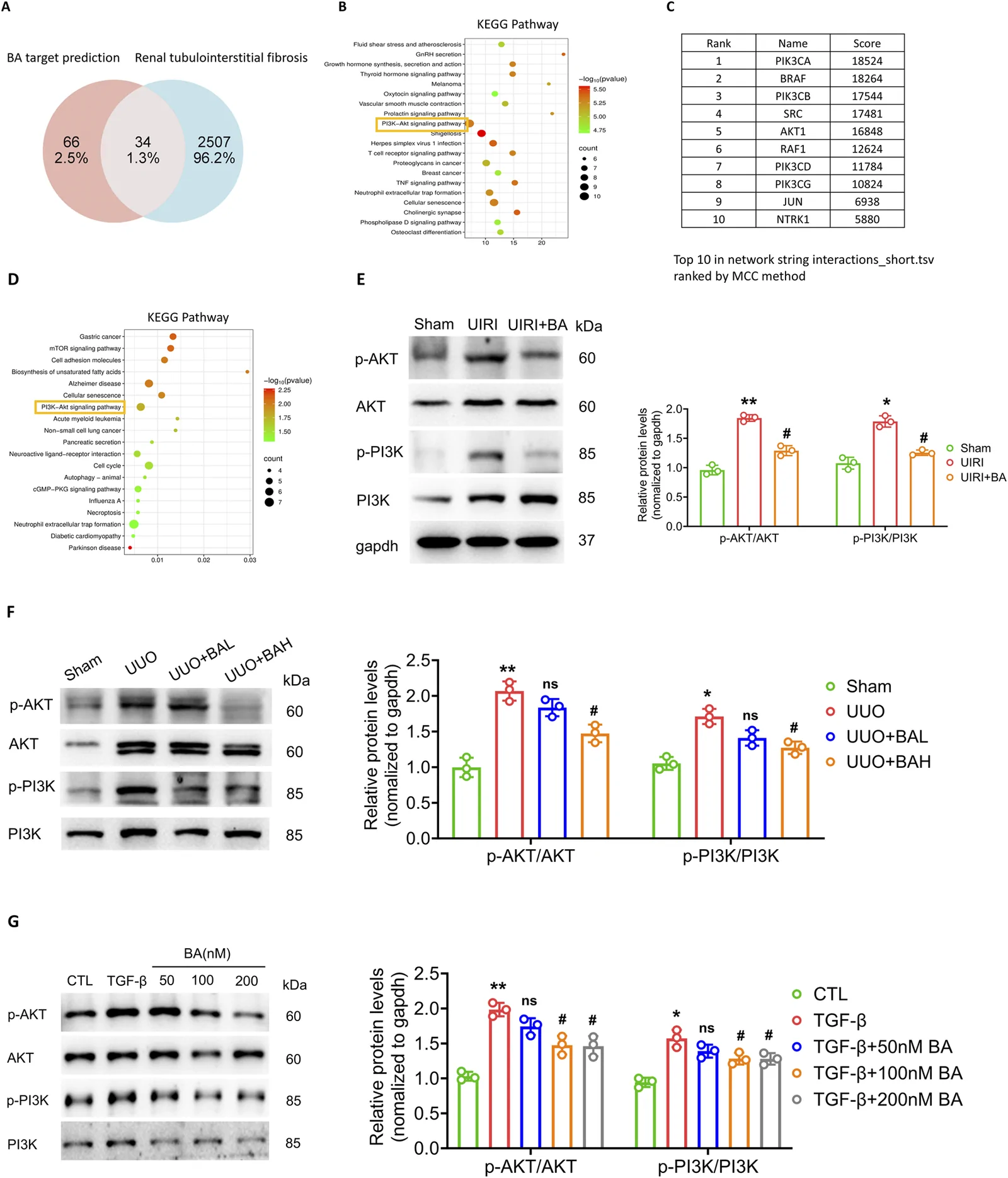
BA suppressed PI3K-AKT pathway activation in fibrotic kidneys from UIRI and UUO mice, as well as in TGF-β1-treated HK-2 cells. **(A)** Venn diagram showing the overlap between predicted BA targets and renal tubulointerstitial fibrosis–related targets. A total of 34 shared targets were identified. **(B)** KEGG pathway enrichment analysis of the 34 shared targets. Bubble size indicates gene count, bubble color represents −log10(P value), and the x-axis represents the number of enriched genes. **(C)** Protein–protein interaction (PPI) network of the top 10 hub genes ranked by maximal clique centrality (MCC), including PIK3CA, BRAF, PIK3CB, SRC, AKT1, RAF1, PIK3CD, PIK3CG, JUN, and NTRK1. PIK3CA ranked first, followed by BRAF, PIK3CB, SRC, and AKT1. **(D)** KEGG pathway enrichment analysis of differentially expressed genes between the UIRI and UIRI + BA groups. **(E)** Representative western blot images and quantitative analysis of phosphorylated PI3K (p-PI3K), total PI3K, phosphorylated AKT (p-AKT), and total AKT protein expression in kidneys from Sham, UIRI, and UIRI + BA groups. Data shown are individual values with means ± SD; n = 3. *P < 0.05, significantly different from Sham group; #P < 0.05, significantly different from UIRI group. **(F)** Representative western blot images and quantitative analysis of p-PI3K, PI3K, p-AKT, and AKT protein expression in kidneys from Sham, UUO, UUO + BA low-dose (BAL), and UUO + BA high-dose (BAH) groups. Data shown are individual values with means ± SD; n = 3. *P < 0.05, significantly different from Sham group; #P < 0.05, significantly different from UUO group. **(G)** Representative western blot images and quantitative analysis of p-PI3K, PI3K, p-AKT, and AKT protein expression in HK-2 cells treated with TGF-β1, with or without BA (50, 100, or 200 nM). Data shown are individual values with means ± SD; n = 3. *P < 0.05, significantly different from CTL group; #P < 0.05, significantly different from TGF-β group. Error bars and exact P values are reported in Supplementary File 1.

To explore the molecular mechanisms underlying the anti-fibrotic effects of BA, network pharmacology analysis was performed. A total of 34 overlapping targets were identified between BA-related targets and renal tubulointerstitial fibrosis-associated genes (Figure 6A). KEGG pathway enrichment analysis revealed significant enrichment of these targets in the PI3K/AKT signaling pathway (Figure 6B). Protein–protein interaction (PPI) network analysis identified the top 10 hub genes, ranked by maximal clique centrality (MCC). Among these genes, PIK3CA was identified as the highest-ranked hub gene. Notably, several hub genes, including PIK3CA, AKT1, and SRC, were closely associated with the PI3K/AKT signaling pathway (Figure 6C). Moreover, KEGG pathway enrichment analysis of differentially expressed RNAs from RNA-seq data comparing the UIRI and UIRI + BA groups also implicated PI3K/AKT signaling (Figure 6D). Western blot analysis further confirmed that UIRI markedly increased PI3K and AKT phosphorylation, whereas BA treatment significantly inhibited their activation (Figure 6E). Similar results were observed in the UUO model and HK-2 cells, further supporting that suppression of PI3K/AKT signaling is a reproducible mechanism underlying the anti-fibrotic effects of BA (Figures 6F,G).

### The Wnt7a/PI3K/AKT pathway contributes to the anti-fibrotic effects of BA in HK-2 cells

**FIGURE 7:**
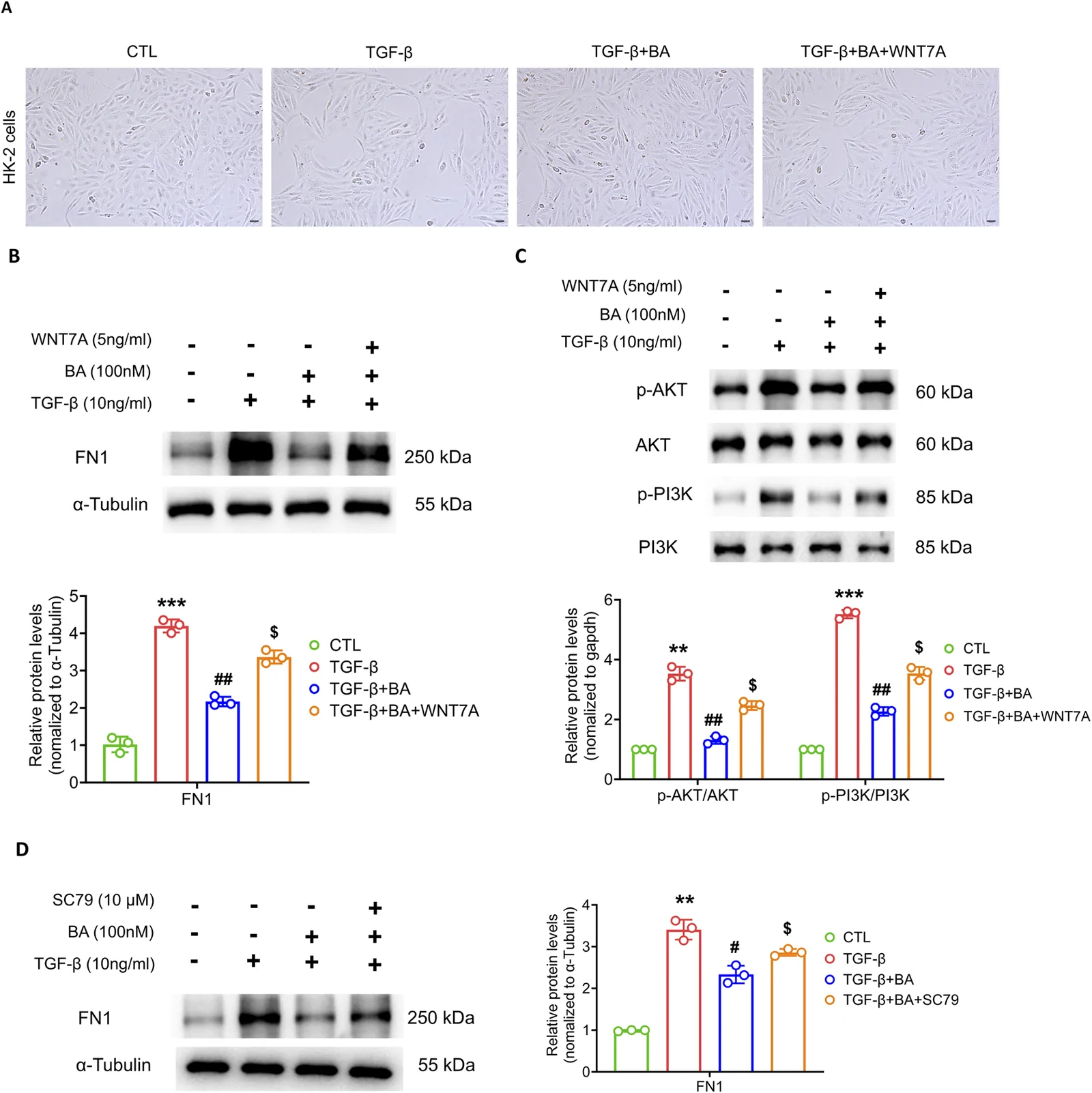
BA inhibited TGF-β1–induced EMT via regulating Wnt7a-PI3K-AKT pathway in HK-2 cells. **(A)** Representative photomicrographs of HK-2 cells in CTL, TGF-β, TGF-β+BA and TGF-β+BA + Wnt7a groups. **(B)** Representative western blot images and quantitative analysis of FN protein expression in HK-2 cells from CTL, TGF-β, TGF-β+BA, and TGF-β+BA + Wnt7a groups. **(C)** Representative western blot images and quantitative analysis of p-PI3K, PI3K, p-AKT, and AKT protein expression in HK-2 cells from the indicated groups. **(D)** Representative western blot images and quantitative analysis of fibronectin (FN) expression in HK-2 cells in CTL, TGF-β1, TGF-β1 + BA, and TGF-β1 + BA+ SC79 (AKT agonist) groups. Data are presented as individual values with mean ± SD (n = 3). *P < 0.05 vs. CTL group; #P < 0.05 vs. TGF-β1 group; $P < 0.05 vs. TGF-β1 + BA group. Error bars and exact P values are reported in Supplementary File 1.

As shown in Figure 7A, TGF-β stimulation induced marked morphological changes in HK-2 cells, characterized by a more elongated and fibroblast-like phenotype, whereas BA treatment partially attenuated these changes. Recombinant Wnt7a protein administration weakened the protective effect of BA. Consistently, TGF-β markedly increased FN1 expression, which was significantly reduced by BA treatment but partially restored after recombinant Wnt7a protein administration (Figure 7B). In addition, western blot analysis demonstrated that TGF-β significantly increased PI3K and AKT phosphorylation, whereas BA suppressed PI3K/AKT pathway activation, as evidenced by decreased p-PI3K/PI3K and p-AKT/AKT ratios. This inhibitory effect was partially reversed by Wnt7a treatment (Figure 7C). Furthermore, the AKT agonist SC79 partially reversed the inhibitory effects of BA on FN protein expression in TGF-β1-treated HK-2 cells (Figure 7D). Collectively, these results suggest that Wnt7a is involved in the anti-fibrotic effects of BA, at least partly through regulation of the PI3K/AKT signaling pathway.

## Discussion

TIF is a common and progressive pathological endpoint of nearly all forms of CKD, characterized by persistent tubular injury, chronic inflammation, myofibroblast activation, and excessive extracellular matrix deposition (Zeisberg and Neilson, 2010; Kang et al., 2015). TIF can be triggered by a variety of injurious insults, including chronic infection, obstructive uropathy, metabolic disorders, autoimmune disease, and nephrotoxic exposure to drugs or heavy metals (Rende et al., 2023). In the present study, we used two classical models of TIF, UUO and UIRI, together with TGF-β1-stimulated HK-2 cells, to assess the protective effects of BA on TIF and elucidate the underlying molecular mechanisms. BA attenuated renal injury and fibrosis in both mouse models and suppressed TGF-β1-induced EMT and fibrogenesis in HK-2 cells. Mechanistically, Wnt7a/PI3K/AKT pathway contributes to the anti-fibrotic effects of BA.

Bruceine A (BA), a natural quassinoid isolated from *B. javanica*, has been reported to possess diverse pharmacological activities, including antitumor, anti-inflammatory, and antiviral effects (Zhang et al., 2021; Sun et al., 2025; Zuo et al., 2025). Increasing evidence also supports its renoprotective potential. Hu et al. showed that BA inhibits galectin-1-mediated inflammatory signaling in rat mesangial cells under high-glucose conditions, suggesting a protective role in diabetic nephropathy (Li et al., 2022). In addition, Li et al. reported that BA attenuates mesangial cell proliferation and inflammation by binding to nuclear receptor 4A1 (NR4A1), thereby exerting protective effects in mesangial proliferative glomerulonephritis (Hu et al., 2025). Based on these two studies demonstrating the glomerular protective effects of BA, in which BA was administered on alternate days at doses ranging from 0.5 mg/kg (low dose) to 2.0 mg/kg (high dose). We initially selected doses of 0.5 mg/kg body weight/day and 1 mg/kg body weight/day in the present study to further investigate its protective effects on renal TIF in the UUO model. BA treatment alleviated UUO-induced histopathological injury and TIF, with more pronounced effects observed at 1 mg/kg body weight/day than at 0.5 mg/kg body weight/day. We then selected 1 mg/kg body weight/day for further validation in the UIRI model, where BA similarly ameliorated renal dysfunction, histopathological injury, and tubulointerstitial fibrosis. UUO and UIRI were used as two well-established models of progressive renal tubulointerstitial fibrosis and tubular injury in the absence of primary glomerular damage (Chevalier et al., 2009; Liang and Liu, 2023). In contrast to previous studies that primarily focused on glomerular protection, particularly mesangial cells, our findings suggest that BA also exerts a protective effect on renal tubulointerstitial injury and fibrosis. In addition to BA, several natural compounds, including formononetin, calycosin, and luteolin, exert anti-fibrotic effects in renal fibrosis through distinct mechanisms involving TGF-β/Smad, PI3K/AKT, NF-κB signaling, MAPKs, inflammation, oxidative stress, and NLRP3 inflammasome activation (Dalal et al., 2025; Singh A. et al., 2026; Singh L. et al., 2026). Although direct comparisons are limited by differences in experimental models, dosing regimens, and outcome measures, BA appeared to exert protective effects at relatively low doses in the present study.

TGF-β1 is widely recognized as a central mediator of renal fibrosis, as it induces epithelial–mesenchymal transition (EMT) and activates multiple profibrotic signaling pathways (Xu et al., 2009; Meng et al., 2016). Accordingly, it is commonly used to establish *in vitro* models that recapitulate key features of renal fibrogenesis (Su et al., 2023). To further determine whether BA exerts a direct protective effect on renal tubular epithelial cells, we performed *in vitro* experiments using TGF-β1–stimulated HK-2 cells. In the present study, BA markedly attenuated TGF-β1–induced morphological changes in HK-2 cells and reduced the expression of fibrosis-related markers, including FN1 and α-SMA. These findings indicate that BA can directly suppress TGF-β1–mediated profibrotic responses in renal tubular epithelial cells. Importantly, these *in vitro* results complement our *in vivo* observations in the UUO and UIRI models, in which BA also alleviated tubulointerstitial injury and fibrosis. Since both UUO and UIRI are classic models characterized predominantly by tubular injury and interstitial fibrogenesis rather than primary glomerular damage, the protective effects observed in HK-2 cells further support the notion that renal tubular epithelium is an important direct target of BA. This is of particular significance because persistent tubular epithelial injury is now considered a major driver of maladaptive repair, myofibroblast activation, and extracellular matrix deposition during the progression of CKD (Qi and Yang, 2018; Schunk et al., 2021; Fu et al., 2022; Li et al., 2024). Taken together, our data suggest that BA alleviates renal fibrosis, at least in part, by directly protecting renal tubular epithelial cells against TGF-β1–driven profibrotic injury.

Wnt7a, a member of the Wnt family, is minimally expressed or absent in healthy adult kidneys but is significantly upregulated in diseased kidneys, particularly in CKD and autosomal dominant polycystic kidney disease (ADPKD) (Qin et al., 2012; Ming et al., 2023). Previous studies have shown that Wnt7a is upregulated in experimental renal fibrosis and that inhibition of Wnt7a/β-catenin signaling alleviates fibrotic injury, supporting its role as an upstream profibrotic ligand (He et al., 2011; Zhou et al., 2013). In the present study, transcriptomic analysis identified Wnt7a as a key differentially expressed gene that was significantly upregulated in the kidneys of UIRI mice and suppressed by BA treatment. This finding was further confirmed at the protein level by western blotting and immunohistochemistry. Notably, Wnt7a expression was mainly localized to renal tubular epithelial cells, particularly proximal tubules, and was markedly increased after UIRI but attenuated by BA treatment. These findings suggest that Wnt7a may be involved in tubulointerstitial injury and fibrosis and that suppression of Wnt7a may represent one of the mechanisms underlying the renoprotective effect of BA.

To further explore the potential mechanisms underlying BA-mediated protection against TIF, we performed network pharmacology analysis. 34 overlapping targets were identified between BA-related targets and renal TIF-associated genes, and KEGG enrichment analysis highlighted the PI3K/AKT signaling pathway. Consistently, PPI analysis identified PIK3CA as the top-ranked hub gene. Although network pharmacology is widely used to predict potential drug targets and explore underlying signaling pathways, this approach has inherent limitations because it relies largely on database-derived information and computational algorithms. Therefore, we further performed RNA-seq analysis, which identified the PI3K/AKT signaling pathway as a key pathway potentially involved in BA-mediated attenuation of renal TIF. Consistent with the transcriptomic and network pharmacology analyses, western blotting further validated that BA inhibited PI3K/AKT pathway activation in fibrotic kidneys from UUO and UIRI mice, as well as in TGF-β1-treated HK-2 cells. In line with our results, several natural compounds, including formononetin, quercetin, luteolin and kokusaginine, have been reported to attenuate renal fibrosis partly through suppression of PI3K/AKT-related signaling (Tu et al., 2021; Wang et al., 2024; Singh A. et al., 2026; Singh L. et al., 2026). Taken together, our findings indicate that the PI3K/AKT signaling pathway may be involved in the protective effects of BA against renal TIF and further support PI3K/AKT inhibition as a potential therapeutic strategy for renal tubulointerstitial injury and fibrosis.

Although Wnt7a has been reported to act as an upstream activator of the PI3K/AKT/mTOR pathway in skeletal muscle (von Maltzahn et al., 2011), its relationship with PI3K/AKT signaling in renal fibrosis remains largely unclear. In this context, our *in vitro* findings support a functional link between Wnt7a and PI3K/AKT signaling in renal fibrotic responses. TGF-β1 induced marked morphological changes in HK-2 cells, accompanied by increased FN1 expression and activation of PI3K/AKT signaling, as indicated by elevated p-PI3K and p-AKT levels. BA largely reversed these profibrotic changes, whereas exogenous Wnt7a partially abolished its protective effects, as evidenced by restoration of fibroblast-like morphology, FN1 expression, and PI3K/AKT activation. These results suggest that Wnt7a may act upstream of PI3K/AKT signaling and contribute to tubular profibrotic responses *in vitro*. Given that tubular epithelial injury is a central event in the initiation and progression of TIF, our findings indicate that BA attenuates renal fibrosis by suppressing Wnt7a-associated PI3K/AKT activation.

Several limitations of this study should be acknowledged. First, although our rescue experiments in TGF-β1-treated HK-2 cells support a potential mediating role of the Wnt7a/PI3K/AKT pathway in the protective effects of BA on renal tubular epithelial cells, this mechanism has not yet been confirmed *in vivo*. Further studies using Wnt7a overexpression, as well as pharmacological or genetic modulation of PI3K/AKT signaling in animal models, are required to further clarify the causal contribution of this pathway to BA-mediated renal protection. Second, although BA is suggested to exert anti-fibrotic effects partly through inhibition of the Wnt7a/PI3K/AKT signaling axis, the PI3K/AKT pathway plays central roles in multiple essential cellular processes, and potential off-target effects cannot be fully excluded. In the present study, no apparent systemic toxicity was observed at the tested doses; however, the long-term safety profile and pharmacological specificity of BA require further investigation. Finally, some western blot analyses were performed with a relatively small number of independent biological replicates (n = 3). Although statistically significant differences were observed and the western blot findings were generally consistent with histological, immunohistochemical/immunofluorescence, transcriptomic, and functional rescue data, the small sample size may limit the statistical power of these experiments. Future studies with larger sample sizes are needed to further strengthen the statistical robustness of these results.

**FIGURE 8:**
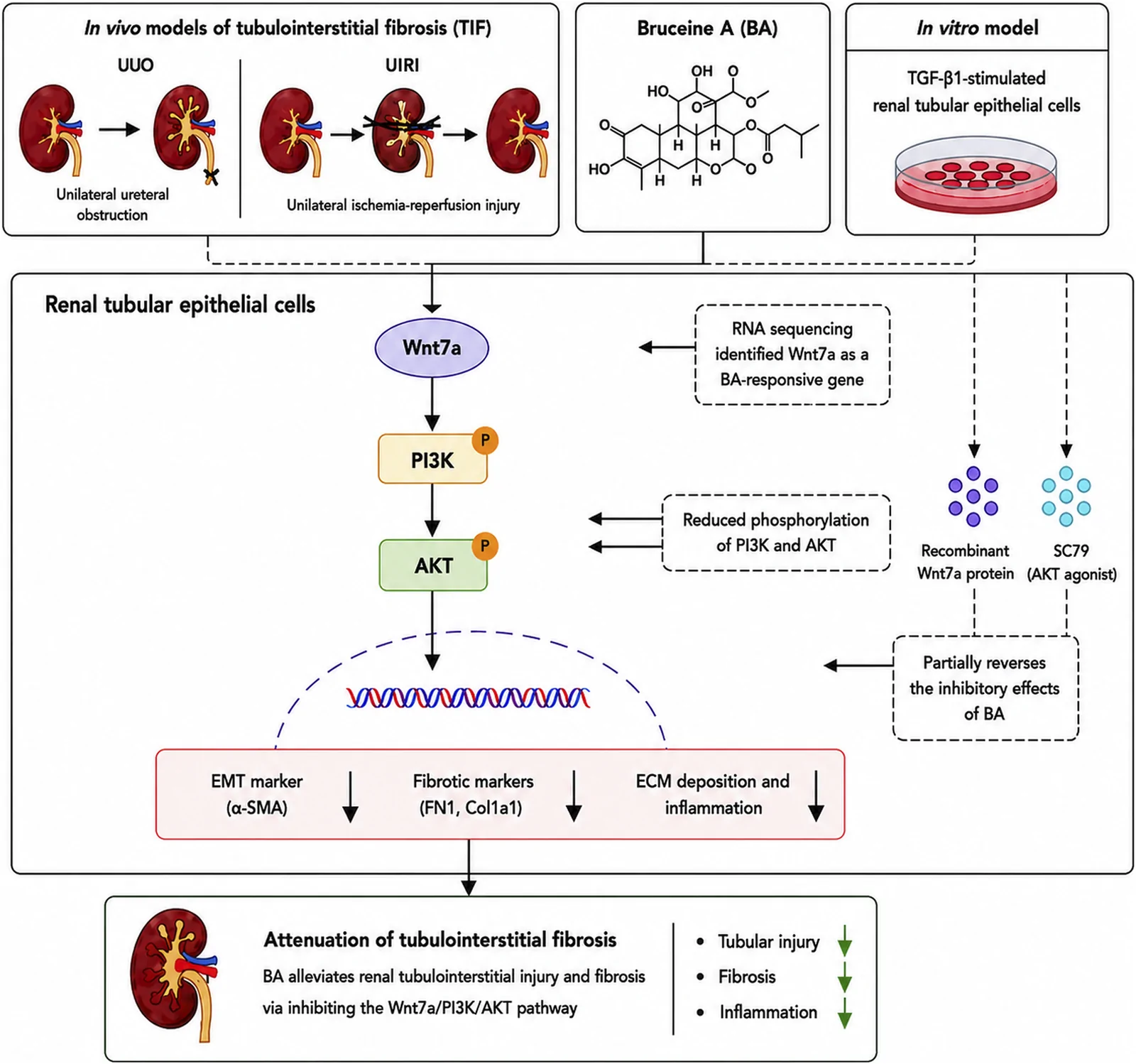
Schematic diagram of the proposed mechanism of BA in TIF. BA attenuates renal tubulointerstitial injury and fibrosis induced by UUO and UIRI, accompanied by downregulation of Wnt7a expression and inhibition of PI3K/AKT pathway activation. Recombinant Wnt7a protein and the AKT agonist SC79 partially reverse the protective effects of BA against TGF-β1-induced fibrogenesis in HK-2 cells. These findings suggest that the Wnt7a/PI3K/AKT pathway contributes to the antifibrotic effects of BA, supporting its potential as a therapeutic candidate for renal tubulointerstitial injury and fibrosis.

In summary, our findings suggest that BA attenuates UUO- and UIRI-induced renal tubulointerstitial injury and fibrosis, accompanied by downregulation of Wnt7a expression and suppression of PI3K/AKT pathway activation. Recombinant Wnt7a protein and the AKT agonist SC79 partially reversed the protective effects of BA against TGF-β1-induced fibrogenesis in HK-2 cells (Figure 8). These findings indicate that the Wnt7a/PI3K/AKT pathway may contribute to the antifibrotic effects of BA, supporting BA as a potential therapeutic candidate for renal tubulointerstitial injury and fibrosis.

## Data Availability

The RNA-sequencing data generated in this study have been deposited in the NCBI Gene Expression Omnibus repository under accession number GSE339176 and are publicly available at [https://www.ncbi.nlm.nih.gov/geo/query/acc.cgi?acc=GSE339176].
