# Supplementary material for: Bruceine A attenuates renal tubulointerstitial fibrosis by inhibiting the Wnt7a/PI3K/AKT pathway
Source: Front Pharmacol. 2026 Sep 7;17:1865261. doi: 10.3389/fphar.2026.1865261 (PMC13590780; doi:10.3389/fphar.2026.1865261)
Supplement: Supplementary file 2 [file Table1.docx]

| **Gene** | **Forward primer (5′→3′)** | **Reverse primer (5′→3′)** |
| --- | --- | --- |
| GAPDH | AGGTCGGTGTGAACGGATTTG | TGTAGACCATGTAGTTGAGGTCA |
| KIM-1 | ACATATCGTGGAATCACAACGAC | ACAAGCAGAAGATGGGCATTG |
| FN1 | ATGTGGACCCCTCCTGATAGT | GCCCAGTGATTTCAGCAAAGG |
| α-SMA | CTGACAGAGGCACCACTGAA | CATCTCCAGAGTCCAGCACA |
| COL1A1 | GCTCCTCTTAGGGGCCACT | CCACGTCTCACCATTGGGG |
| TNF-α | CCCTCACACTCAGATCATCTTCT | GCTACGACGTGGGCTACAG |
| MCP-1 | TTAAAAACCTGGATCGGAACCAA | GCATTAGCTTCAGATTTACGGGT |
| IL-1β | GCAACTGTTCCTGAACTCAACT | ATCTTTTGGGGTCCGTCAACT |
| NGAL | TGGCCCTGAGTGTCATGTG | CTCTTGTAGCTCATAGATGGTGC |

**Table.1 Primer sequences used for qRT-PCR**
