# Supplementary material for: Bruceine A attenuates renal tubulointerstitial fibrosis by inhibiting the Wnt7a/PI3K/AKT pathway
Source: Front Pharmacol. 2026 Sep 7;17:1865261. doi: 10.3389/fphar.2026.1865261 (PMC13590780; doi:10.3389/fphar.2026.1865261)
Supplement: Supplementary file 3 [file Supplementaryfile1.docx]

Error bars and exact P values for all figure panels
